# Occupational Therapy Services in School-Based Practice: A Pediatric Occupational Therapy Perspective from Ireland

**DOI:** 10.1155/2021/6636478

**Published:** 2021-06-16

**Authors:** Carol O'Donoghue, Jennifer O'Leary, Helen Lynch

**Affiliations:** Department of Occupational Science and Occupational Therapy, University College Cork, College Road, Cork, Ireland

## Abstract

**Purpose:**

School is a primary setting for pediatric occupational therapy practice, yet little is known about the provision of school-based occupational therapy in many countries internationally. The purpose of this study was to explore current school-based occupational therapy practice for the first time in Ireland to gain insight into current and potential service provision and to identify new directions and potential pathways for development.

**Methods:**

This descriptive quantitative study utilized a cross-sectional online survey to gain the perspectives of the population of pediatric occupational therapists working regularly in schools across Ireland. Respondents were recruited through convenience and snowball sampling. Data were analysed through qualitative content analysis and descriptive statistics.

**Results:**

The survey elicited 35 responses, yielding a 21.2% estimated response rate. Findings demonstrated that respondents provided therapy services in schools most commonly on a weekly (28.6%) or monthly (34.3%) basis, with only 5.0% working in the same school on a weekly or fortnightly basis. The majority of respondents (54.3%) used a direct therapy approach with a child, rather than coaching or modelling, to primarily address sensory, hand function, or daily living needs. None used a whole class or whole school (universal or tiered) approach. While respondents (54.3%) generally viewed collaborative practice as a strength of school-based practice, they also identified barriers to collaboration in schools. A core barrier is related to how services are constructed across health and education, with differing philosophies of service provision. The majority of respondents (75.0%) reported that they had not received any training to deliver evidence-based practice in therapy provision specific to school-based practice. *Implications for Practice*. This study indicates that therapists require continual education on evidence-based school practice as it applies in an Irish context. Furthermore, clarification of school therapy roles and service delivery models are required in order to determine how they contrast with traditional clinic roles. This will enable therapists to strengthen the coordination of service delivery between health and educational services to maximize the outcomes of school-based practice.

## 1. Introduction

School-based occupational therapy (SBOT) is an area of increasing international attention reflected by the first publication on this topic by the World Federation of Occupational Therapy (WFOT) in 2016. In this document, WFOT established the role of occupational therapists in school-based practice as one that is occupation-based and educationally relevant, by supporting student wellbeing, while also promoting and maximizing participation [1]. Therapists are guided to do this by identifying students' strengths and resources to find solutions and in turn limit or remove challenges in learning. This expands on the American Occupational Therapy Association's (AOTA) guidance on SBOT practice, which emphasizes the use of expertise to enable children to prepare for and engage in significant learning and developmental activities within the school environment [2]. Therefore, SBOT is now more clearly related to enabling participation in school-based occupations overall [3, 4]. Yet, to date, while it is apparent that occupational therapists have a role to play in schools, there is limited evidence as yet on how this occurs in relation to wellbeing and the tools and intervention approaches utilized that enable participation specifically [5].

Therefore, determining the best evidence for working in school settings is a key priority. According to WFOT, educational needs should be addressed in educational settings as an issue of best practice [1], as interventions are most effective when implemented in the natural environment (e.g., [3, 6–12]). For example, in a recent Swiss study, therapists reported that they needed to experience the natural educational environment of the school to understand the impact of the social and physical contexts on the child [13]. Using their professional reasoning, being in the school environment helped therapists to bring in an occupational perspective that complemented the educational system to accommodate the child's needs [13].

Once in the school, the next question is what form of service delivery is most effective? In US studies, it was found that many therapists implement a pull-out approach, removing the student from the classroom to conduct one-to-one direct interventions in therapy rooms (e.g., [7, 8, 10, 14, 15]). In some cases, this is because of teachers' expectations [7] or because therapists have more autonomy over interventions [14], while in Rodrigues and Seruya's study (2019), therapists reported that it was more time-efficient and conducive to student schedules. There are strengths to the pull-out approach; however, many weaknesses have been identified. Christner [10] found that when therapists remove the child from the “natural context” of the classroom, it will inevitably affect their access to the curriculum covered in class which could potentially lead to academic challenges. Instead, she found that there was an improvement in therapy outcomes when performed in the natural environment. Teachers credited the improved outcomes to the presence of therapists in classroom settings which allowed for increased collaborative interventions, which teachers found less disruptive than the more traditional pull-out interventions [10]. Shifting from the pull-out model to an approach involving greater collaboration has been welcomed by therapists in principle, yet this has proven difficult for teaching professionals due to the unfamiliarity of having the therapist in the classroom [14]. Becoming familiar with each other is evidently an important consideration to maximize effectiveness of SBOT.

In recent years, not surprisingly, research has examined more closely the relationship work that underpins SBOT. For example, relationship building and effective collaboration have been identified as core objectives of SBOT [10, 16, 17]. Collaboration is the ability of therapists and colleagues to mutually share expertise and respect each other's unique skills to implement strategies to reach a goal [18]. In an Australian study, teachers identified a range of benefits to collaboration including an increase in students' concentration and a decrease in undesired behaviours [19]. Meanwhile, therapists in Switzerland recognized building collaborative relationships with educators as a significant aspect in developing effective interventions [13]. However, there are barriers to effective collaboration such as lack of time, inability of school staff to carry over strategies, and limited understanding of occupational therapy interventions (e.g., [11, 19]). Additionally, studies have found differing expectations of the role of therapists and teachers in collaborative practices. For example, one study found that teachers believed that therapists did not perceive themselves to be in an equal working relationship [11]. In contrast, in another study, occupational therapists identified that many educators perceived the therapist's role to be consultants, who offer solutions to “fix” a child's challenges, and this too presents a barrier [20]. This general lack of understanding of occupational therapists as part of the team is identified as a common barrier to collaborative practice [8, 10, 11, 19]. Collaboration between these health and education professionals is a complex process that needs ongoing development to be effective [21]. Findings from the literature suggest that therapists need to spend more time in schools to build relationships and develop authentic collaborative practice, in order for their roles to be understood as equal partners rather than experts on the child.

Internationally, occupational therapy services for children traditionally follow a medical model whereby the child is referred, and occupational therapy intervention is provided by the therapist. However, SBOT is beginning to shift away from this form of practice [1, 22]. Instead, other models of service delivery have been developed, such as Partnering for Change (P4C), which uses a needs-based, tiered approach to provide services to enhance occupational participation among the entire school community [17]. Missiuna et al. [17] found that a tiered service delivery model could provide individual students with diverse needs with high intensity therapy, while students at risk could receive targeted group intervention, and the entire school body could receive preventive and proactive interventions. Evaluation of the implementation of the P4C found that this model improved therapists' confidence in delivering a school-based service and allowed for consistency in service provision [23]. Furthermore, the P4C model was effective in eliminating waitlists for occupational therapy as those who required therapy were identified and offered intervention more efficiently in their natural environment [24]. However, this new model of practice brings a challenge to SBOT as therapists have a workload which requires the provision of services to address both special and general education needs [14, 22]. It is unclear yet if tiered models like P4C are transferable to other health and education settings for SBOT delivery internationally.

In regions such as North America, South Africa, and New Zealand, governments have identified the need for therapy in schools and directly employ occupational therapists to work in these contexts. However, therapists in many countries internationally are not employed to work directly in schools resulting in poorly established practices in school settings. This is a consequence of delivering therapy interventions in community-based or private practice settings, with therapists providing school-based services on an irregular basis [11]. Yet, each country has its own history and context for health and education-based occupational therapy provision that results in differing systems of service delivery. For example, in Ireland, occupational therapists worked in special schools in the 20^th^ century, employed by nongovernment organizations primarily [25]. However, practice has since shifted to provide services to a broader range of schools, but based mainly in community clinics, funded by government health departments, which has impacted school practices also [26, 27]. More recently, the Department of Education established a new initiative to pilot an in-school multitiered therapy service [28]. The two-year pilot study began in 2018 by employing 19 speech and language therapists and 12 occupational therapists to provide therapy services for 150 schools and preschools in one region in the east of Ireland [28, 29]. Consequently, the need to establish current SBOT practice from an Irish perspective arose, as there is little research on the current state of SBOT in Ireland overall. Therefore, this research is aimed at contributing to the knowledge gap surrounding SBOT practice in Ireland by exploring the question: what is school-based occupational therapy according to pediatric occupational therapists working in schools in Ireland? The aims of the study were to describe current pediatric occupational therapy service delivery and practices in SBOT and to investigate and explore their knowledge and utilization of evidence-based practice in SBOT.

## 2. Materials and Methods

A quantitative cross-sectional design was selected, using an online survey as this allows for larger sample sizes to be obtained, and thus, data can be gathered that represents a large population at one time [30, 31]. While there are limitations to online data collection such as sample biases and reduced control of the researchers, it is recognized that data generated can still be valid, reliable, and comparable to that of offline studies [32].

### 2.1. Instrumentation

Survey questions were informed by a preexisting survey designed for a SBOT study in Switzerland, which included 52 questions in total and involved a mix of open and closed questions [3]. The authors sought and were given permission to access this survey and to adapt it for use for this Irish study. The survey was critically analysed and adapted by the three coauthors to represent more directly an Irish context and to address the research question for this study. Each question was considered for the contribution it would make to describing as well as understanding SBOT practices, while deleting any that seemed repetitive or redundant. A draft of the amended survey was then sent to the European Network of Therapists in Higher Education School-Based Occupational Therapy (ENOTHE-SBOT) group. Feedback was received from this group and aided the final design of survey questions. Following good practice guidance, a pilot study of the survey was conducted with a local pediatric occupational therapist to further inform the survey development [33]. This feedback informed the final selection, list, number, and order of questions. The final survey contained 34 questions (28 closed questions and six open-ended questions), relating to three core themes: demographics (10 questions), current practice (14 questions), and education (10 questions). Closed questions included a mix of nominal (including multiple choice questions) and ordinal (Likert scales) questions. The open-ended questions related to defining practice, for example, or providing value or attitudinal responses include the following: *From your perspective, how would you define school-based occupational therapy…* or *when working in schools, which interventions do you consider important but do not get to d*o? The open questions strengthened the dataset gathered by elucidating comments that could not be obtained solely using quantitative questions, allowing for answers that may not have been considered or expected [34]. As a result of this process, face validity was addressed by engaging with an expert researcher in pediatric practice who reviewed the survey instrument and confirmed that the survey measured the area of interest of the study [35]. Reliability was addressed through adapting existing survey items and conducting a pilot study, to ensure questions were interpreted appropriately in relation to the research topic [35, 36].

Ethical approval for this research study was granted by the Social Research Ethics Committee, University College Cork, Ireland, in 2018.

### 2.2. Participants

The target population was pediatric occupational therapists who had regular experience of working in schools in Ireland. As therapists are not directly employed in schools in Ireland, the definition used for SBOT in this study was any pediatric occupational therapist working routinely in schools on an average of once a month. School-based occupational therapists were recruited through a convenience sampling method [37] through the Association of Occupational Therapists of Ireland (AOTI) database of members. AOTI emailed members to inform them of the study and invite them to take part via a link to the online SurveyMonkey platform and then recirculated the email after four weeks to maximize the response rate [33]. Potential participants were advised that consent was assumed by submission of the survey, which is common practice for online surveys [38]. To ensure confidentiality of participants, no personal identifying information was requested when completing the survey.

It is estimated that 165 therapists were contacted to participate in the survey. However, neither the total population of pediatric occupational therapists nor the number of these therapists who carry out work in schools in Ireland is known. Therefore, it is only possible to estimate the response rate. In total, 49 survey responses were collected indicating an “actual response rate” of 29.7%. However, of these responses, 14 were marked as incomplete as they only answered the demographics questions (Q.1-10) and so were removed from the dataset prior to analysis. Of the remaining 35 responses, 32 participants completed the survey in its entirety, while 3 participants completed all questions related to demographics and practice but opted out of answering questions relating to training and competence and so were included to maximize the data. The outcome is that the survey generated an “analysable response rate” [33] of 21.2%. This is consistent with the typical estimated response rate of online surveys which is 20% [39]. It is important to note that as many pediatric therapists do not work in schools, this is an underestimate.

### 2.3. Data Analysis

All 35 responses were analysed. Closed questions were analysed using descriptive statistics and presented using pie charts, bar charts, and tables [40]. Open-ended questions were analysed using qualitative thematic content analysis, which supports the researchers to identify, organize, and interpret common perspectives from respondents [41]. Peer review processes were adopted among the three coauthors to strengthen the identification and categorisation of core themes, to enhance credibility, and to maximize rigor. Relationships between qualitative and quantitative data were explored to identify points of triangulation, which served to enhance understanding.

## 3. Results

Results are presented across the three main themes: (a) demographics, (b) description of SBOT practice in school settings, and (c) training and knowledge of SBOT.

### 3.1. Demographics of the Pediatric Occupational Therapists Who Participated in This Study

The full details of the survey respondents' demographics are outlined in Table 1. Survey respondents were predominantly senior grade therapists (*n* = 25, 71.4%), with most therapists having over six years of experience working as a pediatric therapist (*n* = 28, 80.0%). Geographically, Ireland is divided into four provinces. Of these, therapists working in the Munster province make up the majority of respondents (*n* = 24, 68.6%), while no therapists working in Connacht participated. Respondents mainly worked in settings specific to Autism Spectrum Disorder (ASD) and Developmental Coordination Disorder (DCD) (*n* = 24, 68.6%). Many respondents reported working in early intervention settings (*n* = 16, 45.7%) and services addressing physical disability (*n* = 14, 40.0%,), with three respondents working in child and adolescent mental health services (*n* = 3, 8.6%). Respondents predominantly worked with children aged five to seven years (*n* = 17, 45.6%). All respondents noted that they had worked in primary school settings (*n* = 35, 100.0%), with many also having worked in early learning and care centers (*n* = 28, 80.0%), postprimary schools (*n* = 25, 71.4%), and special schools (*n* = 24, 68.6%). Most respondents reported that they worked in more than 10 schools (*n* = 25, 71.4%), with the greatest number of respondents reporting that they work in schools monthly (*n* = 12, 34.3%) or weekly (*n* = 11, 31.4%). Respondents indicated that they work in the same school infrequently, on a needs basis (*n* = 25, 71.4%). Two respondents worked in the same school weekly (*n* = 2, 5.7%), and an additional two respondents worked in the same school biweekly (*n* = 2, 5.7%), while no respondents reported working in the same school daily.

Most respondents reported that they had a waitlist for children to be seen in their service (*n* = 30, 85.7%), with the average waitlist being 12 months. Respondents reported that the length of waitlists ranged from having no waitlist to having a waitlist of up to three and half years.

### 3.2. Describing SBOT in School Settings

Respondents described SBOT as a service that is aimed at “supporting the child to participate in activities in school settings,” “focusing on the needs of the child,” and “focusing on school-based occupations.” Respondents' description of SBOT related to their role; for example, one respondent said, “a school-based occupational therapist addresses the client's needs in a holistic manner,” while another described the role as “someone who is based in schools rather than a clinic.” However, consistent themes emerged among responses, relating to service organization (*n* = 20, 57.1%), focus of intervention (*n* = 20, 57.1%), approach and model (*n* = 10, 28.6%), and consultancy with schools (*n* = 10, 28.6%) which will be explored further.

Respondents acknowledged the role of service organization when defining SBOT, as the service determines how therapists work in or with schools. Some respondents reported that although they carry out some work in schools, they do not see themselves as school-based therapists as they are not exclusively in schools: “I am not a school-based therapist; I come and go to schools where children in my catchment area attend,” while another said, “I am a HSE (health board) employee therefore attend schools by appointment only.” For some, SBOT was based on a system of referrals, whereby “an occupational therapist visits the school as a means of assessment of the referred child.” Furthermore, despite working in schools, others defined SBOT as a service that is only delivered by therapists who are employed by education: “occupational therapists who are based in the school and/or paid by the Department of Education.”

SBOT was also defined based on the focus of intervention. This varied from only addressing educational needs: “remit only over educational needs and needs associated with those,” to only addressing the health needs of the child: “I strongly feel that it is not my role to differentiate or align with the academic curriculum. Teachers have their own training in that. It is my role to support the child in physically accessing/regulating/developing developmental skills, etc.” In general, respondents reported that SBOT was “the same as clinical just school-based.”

Respondents also identified the main needs of students they work with, in delivering SBOT. The most prominent needs were sensory (*n* = 33, 94.3%), hand function (*n* = 32, 91.4%), and activities of daily living (*n* = 24, 80.0%). The majority of respondents reported using sensory interventions (*n* = 31, 88.6%), assistive technology (*n* = 31, 88.6%), environmental adaptations (*n* = 31, 88.6%), and handwriting interventions (*n* = 29, 82.9%). This was consistent with the most frequently used interventions reportedly implemented in schools: sensory strategies (*n* = 25, 71.4%), environmental adaptations (*n* = 21, 60.0%), and handwriting interventions (*n* = 13, 37.1%).

**Figure 1:**
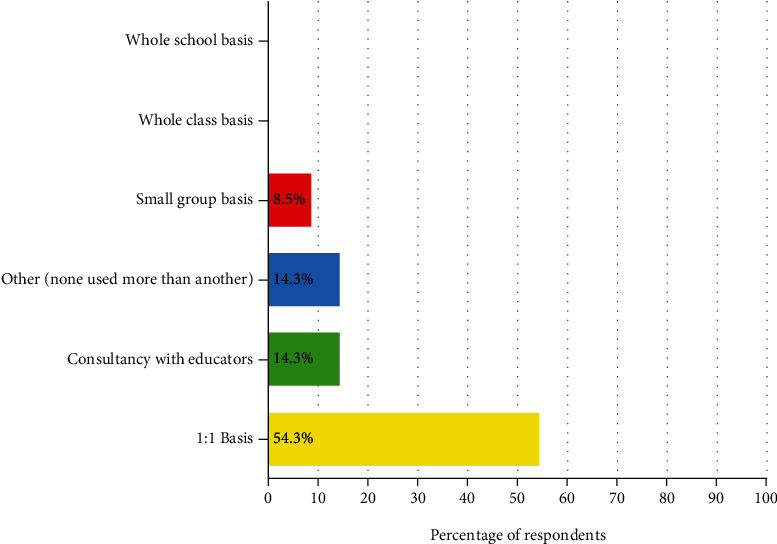
Approach to practice (*N* = 35).

Respondents were asked which approach they used most often when delivering SBOT (see Figure 1). Most respondents used a 1 : 1 approach to practice (*n* = 19, 54.3%). Consultancy with educators (14.3%, *n* = 5) and small group interventions were less common (*n* = 3, 8.5%). However, when asked who they collaborate with in schools, all respondents said educators (*n* = 35, 100.0%) while most said special needs assistants (SNAs) (*n* = 34, 97.1%) and resource teachers (*n* = 33, 94.3%). Some (*n* = 4, 11.4%) respondents identified that they work with teachers by demonstrating or providing strategies and skills to them: “a school-based occupational therapist's role is to support teaching staff to identify strategies that will help the child” or to “demonstrate programs to teachers.” Many (*n* = 6, 17.1%) identified that consultancy meant collaborating and supporting teachers through discussion and observation: “working as part of the teaching team.”

No respondents reported that they worked on a whole class or whole school basis (i.e., a universal approach). However, many discussed the value of a universal approach: “which serves the whole needs of the school, i.e., the school is the client, rather than individual children,” rather than the 1 : 1 approach used in clinical settings. Despite this focus on a 1 : 1 approach to service delivery primarily, respondents recognized the broader impact of SBOT: “working in schools enables the therapist to support children who just need extra exposure and practice with certain tasks, not necessarily due to a disability.”

**Figure 2:**
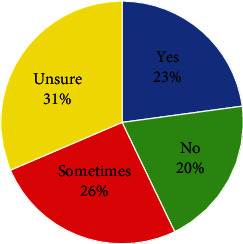
Therapist's responses on their awareness of the consistency between their SBOT practice and other therapists (*N* = 35).

Respondents also determined whether they knew if their school-based practice was consistent with that of other services (see Figure 2). Most reported that they were unsure if their practice was consistent with that of other services (*n* = 11, 31.4%); however, 20% (*n* = 7) said no. One respondent commented: “it is hard to define (SBOT) as every therapist is different.”

**Figure 3:**
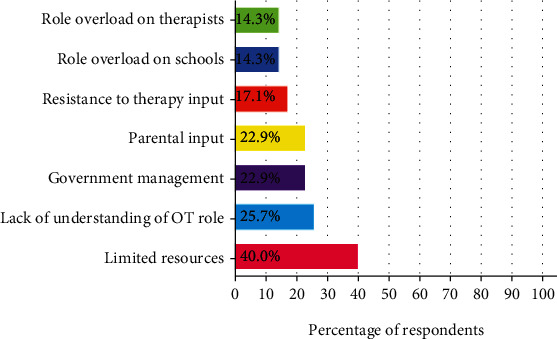
Barriers to SBOT practice.

All respondents believed that there were strengths to SBOT (*n* = 35, 100.0%). Strengths included consulting with educators (*n* = 19, 54.3%), working in the natural environment of the child (*n* = 16, 45.7%), efficiency of the service (*n* = 8, 22.9%), and the universal approach (*n* = 4, 11.4%). However, most respondents believed that there are also barriers to SBOT (*n* = 33, 94.3%), such as lack of time to consult with teachers, alongside the role overload on therapists: “While we hold a dual role of home and school, it is challenging to prioritize all the needs at school,” and educators: “Teachers often cannot sit in on (observe) sessions as they are busy with other children. Teachers have an academic responsibility, so priority is not ‘play or independent living skills.'” Many respondents identified that there are inadequate resources to support SBOT (*n* = 14, 40.0%). All barriers identified are summarized in Figure 3.

### 3.3. Pediatric Occupational Therapists' Training and Knowledge on SBOT

Respondents were asked what training or sources of knowledge informed their therapy practice for SBOT, and the majority of respondents reported that they had not received specific training to work in schools (*n* = 24, 75.0%). When asked what has informed their SBOT, a range of sources were indicated (see Table 2). The most common source of knowledge was continuing professional development (CPD) (*n* = 28, 87.5%) or their occupational therapy degree education (*n* = 21, 65.6%). With regard to CPD, eight respondents reported they had additional training, such as specific sensory interventions and handwriting (*n* = 8, 25.0%). Two respondents reported attending once-off workshops specific to SBOT (*n* = 2, 6.3%).

When asked if there is adequate training available to therapists to work in schools, responses were divided among the 32 respondents who responded to this question. Of the respondents, 50.0% (*n* = 16) agreed that there was adequate training available, while 31.3% (*n* = 10) disagreed. All other respondents neither agreed nor disagreed (*n* = 6, 18.8%). Some respondents elaborated on additional training they would like to receive, including specific SBOT training courses (e.g., P4C) (*n* = 4, 12.5%), training on the role of the teacher (*n* = 4, 12.5%), and training on curriculum accessibility (*n* = 3, 9.4%). However, barriers to training were identified including lack of funding (*n* = 16, 50.0%), lack of time (*n* = 5, 15.6%), and the limited availability of training (*n* = 3, 9.4%).

Likert scales were utilized to establish the confidence levels of respondents when practicing in schools. Most of the 32 respondents reported that they were confident in providing EBP in schools (*n* = 19, 59.4%), collaborating with teachers (*n* = 28, 87.5%), assessing a child in the classroom/school setting (*n* = 26, 81.2%), and identifying student difficulties within the classroom (*n* = 27, 84.3%). When rating their confidence in designing interventions relevant to the educational curriculum, 50.0% (*n* = 16) of respondents reported that they were confident, with 50.0% (*n* = 16) reporting that they were somewhat or not so confident.

Finally, 32 respondents identified changes that they would like to see to SBOT in Ireland. While these changes varied, prominent themes emerged including therapists to be based in schools either full time or more frequently (*n* = 18, 56.3%), increased communication between professionals in schools (*n* = 8, 25.0%), more resources (*n* = 4, 12.5%), and a tiered approach that goes beyond addressing diagnoses (*n* = 3, 9.4%).

## 4. Discussion

This is the first quantitative study conducted in the Irish context aimed at understanding SBOT practice from the pediatric occupational therapist perspective. Respondents represented different practice contexts and have worked in a variety of school settings with children of varied needs, allowing rich perspectives to be gathered. From the respondents in this study, all were working in different schools on a monthly basis, with the majority covering more than 10 schools in their allocation each month. Few worked daily, weekly, or monthly in the same school, but instead, the most common allocation was to visit a school once a month while some visited schools weekly, on a needs-led basis.

Although pediatric therapists in Ireland work in diverse contexts across health and education, they are not employed directly in schools. The impact of this service organizational structure was a key theme in the survey. For example, some therapists suggested that SBOT practice is only for therapists who are allocated to work in a school(s) full-time. Others clarified that while they conducted school visits, they did not consider themselves a “school-based” practitioner as their primary workplace was in the community. This appeared to influence the focus of intervention consequently, with some believing that SBOT was strictly for addressing health and development needs, while others felt that SBOT should address all concerns including educational needs relating to the child's participation in school. These differing perspectives were often at odds with the educators who prioritized academics in contrast to the occupational therapists who focus on health. Similar diversity in opinions about what constitutes SBOT has been found in other school-based studies in Ireland, UK, and Greece, for example, where the source of employment across sectors added additional barriers and complications to providing consistent services (e.g., [21, 42, 43]). The resultant lack of clarity in SBOT practice in Ireland led to therapists in this study working in different ways with many viewing SBOT as the same as clinic-based practice but in a different setting. This is a troubling issue as SBOT has its own sources of evidence for effectiveness and good practice, which need to be considered. In the US, this challenge has been overcome as national and local guidelines to school-based service provision have been established [2, 44]. Yet, in Ireland, other than the guidelines published by the NCSE [28] specific to their pilot project, there are no guidelines for pediatric occupational therapists working in schools, limiting the possibility of benchmarking across services. Further work is needed to establish national guidelines in SBOT irrespective of the source of employment, to ensure that practitioners have the necessary guidance on what constitutes best practice in SBOT.

Respondents in this study described SBOT primarily as a traditional referral and direct intervention model of service delivery. The most commonly used interventions reported by respondents were remedial in nature and primarily delivered by the therapist to individual children on a 1 : 1 basis (handwriting and sensory interventions). This is similar to findings in USA, Canada, and Switzerland, where therapists frequently implement remedial interventions in schools [3, 23, 45]. However, remedial interventions require a significant amount of time and repetition to be effective [46]. For example, Hoy et al. [47] explored a variety of remedial handwriting interventions and found that they were effective when carried out at least twice per week over 20 sessions. Evidence supports that therapists need to be working with the child more frequently or else collaborating with educators to carry over these strategies for these remedial interventions to be effective [48]. Yet, in this current study, 71.4% of respondents reported that they worked in the same school infrequently, with no respondents reporting that they worked in the same school daily. Given the challenges of working within services with high waitlists, and the consequence of having limited time to provide therapy interventions, it is not surprising that respondents were unable to deliver more frequent interventions. However, from this data, it is difficult to know what intervention approaches specifically were being implemented. Further research is needed to examine and differentiate the diverse approaches being used in the Irish SBOT context and the expected outcomes for SBOT in general, especially in relation to the effectiveness of outcomes for social participation and inclusion [5, 49, 50].

Although most respondents are implementing a 1 : 1 approach, collaboration was identified by 54.3% of respondents as a particular strength of school-based practice, similar to international SBOT perspectives [10, 17], with the majority of respondents (87.5%) indicating a high level of confidence in their collaboration skills. However, conversely, collaboration was not identified as a particular strength of SBOT by 45.7%, and only 14.3% of respondents reported working using collaboration as a primary approach, so it appears that there is a discrepancy between what therapists value and what they are able to implement. This is likely due to the lack of time therapists were able to spend in each school and the infrequent attendance in each school setting, which has been acknowledged as a fundamental requirement to implementing effective SBOT [10, 19, 23]. There were also differences in application of collaborative consultation. Some respondents viewed SBOT as a collaborative practice with the teaching team as equal partners, while others believed that school-based therapists should take a consultancy approach, solely demonstrating strategies to teachers. From this data, it could be interpreted that different approaches were being adopted by therapists for different situations or as a response to teacher expectations similar to other studies (e.g., [20]). Alternatively, it may also be an indicator of a lack of training and understanding of collaborative consultation as a different way of practice. Interestingly, no respondents reported that they had received training on school-based collaboration or consultation and only 12.5% of respondents expressed this as a training need.

So, where did the therapist learn about SBOT practice? Most respondents reported that they did not have specific training to work in schools and were applying the skills gained from other CPD such as handwriting programs or sensory integration theory to inform their practice in the school context. In addition, they were gaining their knowledge of SBOT primarily from other sources such as academic papers and work colleagues. Overall, respondents reported a lack of available courses to meet their needs. The challenge of ensuring therapists receive education for SBOT, evidence-based practice, and knowledge translation has been noted as a significant issue internationally also. Best evidence should inform the practices of occupational therapists in every setting, including SBOT [12]. In Szucs et al.'s USA SBOT study, researchers found that therapists were not employing EBP due to time constraints, limited access to evidence, and a lack of understanding among therapists on how to apply the evidence [12]. In another US-based study, researchers identified that therapists required more support to apply EBP in schools, and peer mentoring was explored as a possible solution to address this need [8]. Consequently, it is clear that therapists require further education and support to apply EBP in schools as a unique setting of practice. In P4C, one of the key characteristics for successful implementation was the provision of mentoring and modules to the practitioners to support implementation and to enhance EBP skills and knowledge among therapists [51]. In this Irish study, respondents identified a lack of available training and education as a deterrent to the provision of school-based therapy, with only an emerging awareness of the evidence-based for SBOT as compared to clinic-based practice. Hence, SBOT practitioners in Ireland would benefit from additional educational support, such as the development and provision of specific CPD education on SBOT and including peer mentoring or journal clubs, to enhance good practice.

This is a special moment in time for SBOT in Ireland due to the new in-school therapy project, which was ongoing during the period that this research study took place. It is hoped that from this work will emerge a new direction in service delivery models for SBOT. It is evident from critical reviews of service delivery models, that they are necessary to guide best practice in SBOT (e.g., [1, 14, 17, 23, 52–54]). Ongoing commitment to EBP and research is required both within the profession and across education and health to evaluate how pediatric occupational therapists may utilize and apply these models to guide their approach to practice in schools in Ireland.

### 4.1. Implications for Future Practice and Research

Following this study, a number of implications for future SBOT practice and research arose. These included the following:

- SBOT requires ongoing research as it is an emerging area for the profession in Ireland. A mixed-method research study on perspectives of pediatric occupational therapists working in schools in Ireland would be beneficial to gain further insight into therapists' personal experiences of working in schools and a more comprehensive understanding of their level of training around school-based practice
- Consistent with findings of previous SBOT studies, it was found that therapists lack awareness and understanding of how to employ an evidence-based service in school settings. It would be beneficial for future research to be conducted on the development of training for the provision of a SBOT service
- While this study is aimed at identifying the position of SBOT practice in Ireland from the perspectives of pediatric occupational therapists, research is yet to be conducted from the perspectives of educators in Ireland. This research could further assist in the understanding of SBOT practice and the development of training to assist collaborative practice between professionals

### 4.2. Limitations of the Study

While this is the first quantitative study on current SBOT in Ireland, there were limitations which impact the study's generalizability. Firstly is the number of pediatric occupational therapists who conduct work in schools, and therefore, met survey inclusion criteria are unknown. The researchers aimed to achieve the average response rate of online surveys, which is 20% [39], and were successful in achieving this goal. While a higher response rate may have assisted in ensuring that the population was effectively represented, there is no longer a precise number that determine what an acceptable response rate is [55]. It is now recognized that the quality of respondents in meeting inclusion criteria may be preferable to the quantity of respondents to maintain a study's validity [55]. The majority of respondents were senior grade therapists, with a minimum of six years of experience. This ensured the data collected was from therapists with a depth of practical experience to draw from to answer the survey.

Although the study is aimed at representing pediatric occupational therapists across all of Ireland, there were no respondents from the Connacht region. While this impacts the national representation of the study, every effort was made to maximize response rates through the study's gatekeeper. Lastly, this survey was conducted during the COVID-19 pandemic, which has impacted therapists' work. Many therapists have been redeployed to other areas during this period and so may have had limited access to their professional emails through which they would have received the invitation to the survey.

## 5. Conclusions

Despite much international research on the benefit of SBOT, it is yet to be formally established in mainstream schools nationally in Ireland. Yet, similar to other jurisdictions, many therapists are currently employed by health, to work across health and education sectors in Ireland, and are working in SBOT on a monthly basis. This cross-sectoral employment system was viewed by the therapists as a significant barrier to SBOT, due to the challenges of working intersectorally with differing organizational expectations. This finding is a significant concern in the Irish context and highlights a need for the design and delivery of therapy services to be reviewed.

From a SBOT perspective, this study's findings highlight that while pediatric occupational therapists appreciate the benefits of practicing in the child's natural educational environment, they may not have sufficient knowledge and training to implement SBOT as an evidence-based intervention in schools. Although some consistency was visible in therapists' school-based practices, there were varied opinions and perspectives of what characterizes SBOT among this population group. There was also uncertainty among therapists about whether their SBOT practice was consistent with that of other therapists in Ireland. This highlights a need to establish national guidelines and training to ensure that equitable services are provided to all children. One way to start is to adopt the WFOT document to guide the way forward, towards new ways to enhance child school-based participation from a strengths-based, collaborative, educational-centered, but occupational-based model of practice.

## Figures and Tables

**Table 1:** Demographics of pediatric occupational therapists who participated in this study.

Characteristic (*N* = 35)	*n* (%)
Grade of therapist	
Senior grade	25 (71.4)
Basic grade	8 (22.9)
Manager	1 (2.9)
Clinical specialist	1 (2.9)
Length of time working as a pediatric occupational therapist	
6+ years	28 (80.0)
3-5 years	6 (17.1)
1-2 years	0 (0.0)
Less than a year	1 (2.9)
Location of practice	
Munster	24 (68.6)
Leinster	10 (28.6)
Ulster	1 (2.9)
Connacht	0 (0.0)
Area of pediatric practice	
Early intervention	16 (45.7)
Physical disability	14 (40.0)
Intellectual disability	9 (25.7)
Child and adolescent mental health	3 (8.6)
Other (e.g., ASD and DCD)	24 (68.6)
Age group of children therapists usually work with	
0-4 years	9 (25.7)
5-7 years	17 (45.6)
8-10 years	7 (20.0)
11-13 years	1 (2.9)
14+ years	1 (2.9)
School settings therapists have worked in	
Primary schools	35 (100.0)
Early learning and care centers	28 (80.0)
Postprimary schools	25 (71.4)
Special schools	24 (68.6)
Numbers of schools in therapists' workload	
>10	25 (71.4)
7-9	2 (5.7)
4-6	6 (17.1)
2-3	2 (5.7)
1	0 (0.0)
How often therapists work in schools, in general	
Monthly	12 (34.3)
Weekly	11 (31.4)
Biweekly	4 (11.4)
Daily	0 (0.0)
Needs dependent, infrequently	8 (22.9)
How often therapists work in the same school	
Monthly	6 (17.1)
Weekly	2 (5.7)
Biweekly	2 (5.7)
Daily	0 (0.0)
Other (e.g., needs dependent and infrequently)	25 (71.4)

**Table 2:** Source of knowledge for school-based practice (*N* = 32).

Source of knowledge (*N* = 32)	*n* (%)
CPD learning	28 (87.5)
Occupational therapy degree	21 (65.6)
Practice of colleagues	20 (62.5)
Academic research papers	20 (62.5)
Practice of other professionals	17 (54.3)
Trial and error	17 (54.3)
Textbooks	13 (40.6)
Masters learning	7 (21.9)
Social media outlets	5 (15.6)
P4C	4 (12.5)
Nonrespondents	3 (8.6)
Other, e.g., practice abroad	1 (2.9)

## Data Availability

Due to the data being personal and given under a promise of anonymity, data used to support the findings of this study are not available on open access. However, supplemental material such as the survey is available on request from the authors.
